# Malakoplakia of the small intestine presenting as an abdominal mass

**DOI:** 10.1093/jscr/rjad712

**Published:** 2023-12-30

**Authors:** Nazar Akhverdyan, Marina Mutter

**Affiliations:** School of Medicine, University of Colorado, Aurora, CO 80045, United States; Division of Hospital Medicine, University of Colorado School of Medicine, Aurora, CO 80045, United States

**Keywords:** malakoplakia, abdominal mass, Michaelis–Gutmann bodies, von Hansemann cells

## Abstract

Malakoplakia is a rare chronic granulomatous disease that typically involves the urinary and gastrointestinal tracts of immunocompromised individuals. Characteristic histologic features include von Hansemann cells and Michaelis–Gutmann bodies. Clinical manifestations, based on the organ system effected, range from cutaneous lesions, irritative urinary symptoms, and hematochezia. We report a rare example of malakoplakia presenting as an abdominal mass with extensive intestinal and pelvic involvement complicated by a superficial polymicrobial abscess. This case report aims to describe the proposed pathogenesis, variable clinical presentation, and surgical management of malakoplakia.

## Introduction

Malakoplakia is a chronic granulomatous disease caused by defects in macrophage bactericidal function that leads to impaired digestion of phagocytized microorganisms [1]. Specifically, the proposed pathogenesis is thought to involve impaired microtubule function due to decreased beta-glucuronidase and cyclic guanosine monophosphate [1]. Causative organisms include *Escherichia coli*, *Klebsiella*, and *Proteus* [1]. The condition is rare with fewer than 500 cases reported annually in the United States [2]. It preferentially occurs in immunocompromised patients with HIV/AIDS, organ transplantation, malignancy, poorly controlled diabetes, or autoimmune disorders [3, 4]. While malakoplakia has been described in many organ systems, it frequently effects the urinary and gastrointestinal tracts with clinical manifestations ranging from hematochezia and irritative urinary symptoms to cutaneous lesions. Gastrointestinal tract involvement has been associated with colon adenocarcinoma [5, 6]. We report a rare example of malakoplakia presenting as an abdominal mass with extensive intestinal and pelvic involvement complicated by a superficial polymicrobial abscess and cutaneous fistula.

## Case report

A 74-year-old female with a past medical history of hypertension, hyperlipidemia, type 2 diabetes mellitus, and Hurthle cell carcinoma status postthyroidectomy with presumed pulmonary recurrence on surveillance presented with several months of unintentional weight loss and a painful right lower quadrant mass. Review of systems was negative for infectious symptoms, vomiting, diarrhea, or obstipation.

**Figure 1:**
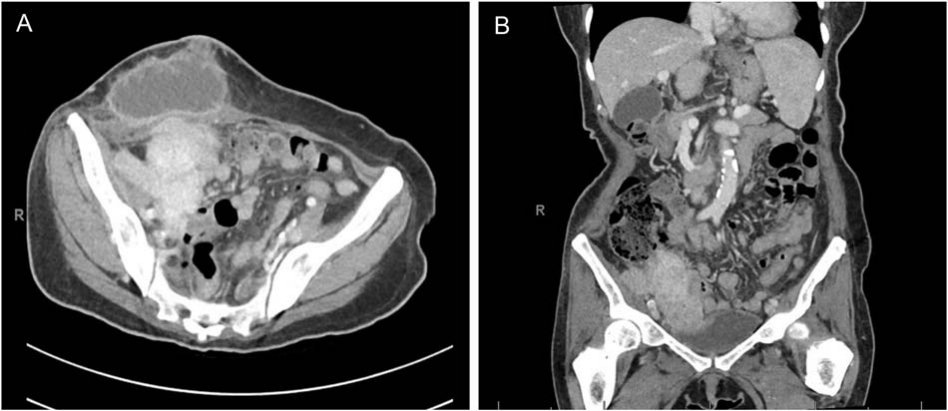
Computed tomography of the abdomen and pelvis in the axial (A) and coronal (B) planes demonstrating a 7.8 × 4.3 cm mass along the right pelvic sidewall and necrotic mass in the anterior abdominal wall with a cutaneous fistula.

Physical exam was notable for diffuse abdominal tenderness to palpation and an indurated right lower quadrant mass with surrounding warmth and erythema. Laboratory studies on admission included a white blood cell count of 16.2 × 10^9^/L with 92% neutrophils. Blood and urine cultures were negative. Computed tomography of the abdomen and pelvis demonstrated a 7.8 × 4.3 cm right lower quadrant mass abutting the right pelvic sidewall and a superficial fluid collection in the anterior abdominal wall with a cutaneous fistula (Fig. 1). Due to initial concern for malignancy, a biopsy was obtained at an outside hospital that demonstrated confluent sheets of histiocytes with eosinophilic cytoplasm (von Hanseman cells) and intracytoplasmic periodic acid-Schiff positive basophilic inclusions (Michaelis–Gutmann bodies), consistent with malakoplakia.

The patient was then transferred to our hospital for a second pathologic opinion as well as surgical and infectious disease consultations; the diagnosis of malakoplakia was confirmed by a review of the pathologic specimens. There was no evidence of malignancy. The abscess began spontaneously draining during admission and cultures grew *E. coli*, *Proteus mirabilis*, *Bacteroides fragilis*, and *Candida glabrata*. She was treated with a 4-week course of trimethoprim-sulfamethoxazole, amoxicillin-clavulanate, and fluconazole. One month later, exploratory laparotomy revealed that the mass invaded the terminal ileum, cecum, and right ovary. Resection with en-bloc ileocecectomy, right oophorectomy, and right ureteral stent placement was performed. Postoperative course was uneventful, and the patient exhibited significant clinical improvement on follow-up.

## Discussion

The nonspecific and variable presentation of malakoplakia can delay diagnosis and treatment. It can be included in the differential diagnosis for malignancy, sarcoidosis, tuberculosis, Crohn’s disease, disseminated fungal infections, and histiocytic disorders [1]. This patient’s radiographic findings and unintentional weight loss were initially concerning for malignancy. The presence of von Hansemann cells and Michaelis–Gutmann bodies on histology aids in diagnosis but is not necessary, especially early in the disease process. While standardized guidelines have not been established given its rarity, treatment involves prolonged antibiotic therapy, surgical resection, and cessation of immunosuppressive therapy when appropriate [1]. Multidisciplinary management is often needed, in this case necessitating close collaboration between pathology, infectious disease, general surgery, urology, and gynecologic oncology. Clinicians should consider malakoplakia in the differential diagnosis for an abdominal mass, especially in those who are immunocompromised.
